# Precise modulation and use of reactive oxygen species for immunotherapy

**DOI:** 10.1126/sciadv.adl0479

**Published:** 2024-05-15

**Authors:** Xinyan Li, Jingjing Gao, Chengcheng Wu, Chaoyu Wang, Ruoshi Zhang, Jia He, Ziting Judy Xia, Nitin Joshi, Jeffrey M. Karp, Rui Kuai

**Affiliations:** ^1^ School of Pharmaceutical Sciences, Tsinghua University, Beijing 100084, China.; ^2^ Tsinghua-Peking Center for Life Sciences, Beijing 100084, China.; ^3^ Department of Anesthesiology, Perioperative, and Pain Medicine, Brigham and Women’s Hospital, Harvard Medical School, Boston, MA 02115, USA.; ^4^ Department of Biomedical Engineering, Material Science and Engineering Graduate Program and The Center for Bioactive Delivery-Institute for Applied Life Sciences, University of Massachusetts, Amherst, MA 01003, USA.

## Abstract

Reactive oxygen species (ROS) play an important role in regulating the immune system by affecting pathogens, cancer cells, and immune cells. Recent advances in biomaterials have leveraged this mechanism to precisely modulate ROS levels in target tissues for improving the effectiveness of immunotherapies in infectious diseases, cancer, and autoimmune diseases. Moreover, ROS-responsive biomaterials can trigger the release of immunotherapeutics and provide tunable release kinetics, which can further boost their efficacy. This review will discuss the latest biomaterial-based approaches for both precise modulation of ROS levels and using ROS as a stimulus to control the release kinetics of immunotherapeutics. Finally, we will discuss the existing challenges and potential solutions for clinical translation of ROS-modulating and ROS-responsive approaches for immunotherapy, and provide an outlook for future research.

## Teaser

Precise modulation and use of reactive oxygen species are critical for safe and effective immunotherapy.

## INTRODUCTION

To maintain homeostasis, cells strictly regulate the production and clearance of reactive oxygen species (ROS), such as hydrogen peroxide (H_2_O_2_), singlet oxygen (^1^O_2_), hydroxyl radicals (·OH), and superoxide anions (O_2_·−) (*1*, *2*). These chemically reactive molecules are by-products of normal oxygen metabolism and can serve as signaling molecules due to their ability to rapidly respond to different stimuli. While excess ROS can trigger harmful oxidative stress, cells can also use this stress as a defense mechanism against pathogens. For example, phagocytes respond to viral or bacterial infections by elevating ROS levels, which serve as a potent mechanism for eliminating these pathogens (*3*, *4*). Inspired by this phenomenon, several ROS-generating biomaterials have been developed to combat viruses, bacteria, and even cancer cells, which are vulnerable to increased ROS levels due to their reduced antioxidant enzyme activity (*1*) compared to healthy cells (*5*, *6*).

More than directly killing invasive cells, ROS can also shape the host immune response against future attacks in various ways. For instance, ROS-mediated killing of pathogens or cancer cells indirectly affects the immune system through the release of pathogen-associated molecular patterns (PAMPs) or damage-associated molecular patterns (DAMPs). Furthermore, ROS can directly affect the functions of immune cells, such as by activating them via increased ROS concentrations (*7*). ROS levels can thus be modulated to directly activate or suppress the functions of different immune cells, including dendritic cells (DCs) (*8*), macrophages (*9*), and T cells (*10*), which holds great promise to improve the therapeutic outcome of cancer and autoimmune diseases. The “ROS-Modulating Strategies to Enhance Immunotherapies” section of this review will thus discuss recently developed biomaterial-based approaches for modulating ROS levels to enhance the efficacy of immunotherapies in the context of infectious diseases, cancer, and autoimmune diseases (Table 1).

**Table 1.** Clinical translation of ROS-modulating strategies. DS, denture stomatitis; DC, dental caries; WNV, West Nile virus; AKs, actinic keratoses; ALA, aminolevulinic acid; MAL, methyl aminolevulinate; NSCLC, non–small cell lung cancer; BCC, basal cell carcinoma; GBM, glioblastoma multiforme; IBD, inflammatory bowel diseases; MS, multiple sclerosis; T1D, type I diabetes; AR, allergic rhinitis; DM, diabetes mellitus; BA, bronchial asthma; AA, allergic asthma; BTM, β-thalassemia major; RA, rheumatoid arthritis; FDA, U.S. Food and Drug Administration.

General indications	Applications	ROS-modulating agents	Mechanism of action	Administration routes	Clinical status
Infectious diseases	DS	TiO_2_ nanoparticles	Photosensitizer	Topical	NCT03666195
	DC	Methylene blue	Photosensitizer	Topical	NCT02908789
	WNV infection	H_2_O_2_-inactivated vaccine (HydroVax-001)	H_2_O_2_ inactivates viruses	Intramuscular	Phase 1
					NCT02337868
	COVID-19	Porphyrins plus sunlight	Photosensitizer	N/A	Phase 1
					NCT04371822
Cancer	AKs	AMELUZ (5-ALA·HCl)	Photosensitizer	Topical	FDA approved
	AKs	LEVULAN KERASTICK (5-ALA·HCl)	Photosensitizer	Topical	FDA approved
	AKs	METVIXIA (MAL)	Photosensitizer	Topical	FDA approved
	NSCLC	PHOTOFRIN	Photosensitizer	Intravenous	FDA approved
		(porfimer sodium)			
	BCC	METVIXIA (MAL)	Photosensitizer	Topical	Phase 3
					NCT00472108
	NSCLC	Topotecan	Radiosensitizer	Oral	Phase 2
					NCT00043862
	GBM	NVX-108 (dodecafluoropentane)	Radiosensitizer	Intravenous	Phase 1
					NCT02189109
	Blood cancer	ZIO-101 (darinaparsin)	Chemical ROS inducer	Intravenous	Phase 1
					NCT00592046
Autoimmune/inflammatory diseases	IBD	Pentoxifylline	Antioxidant	Oral	Phase 2
					NCT05558761
	MS	Idebenone (Raxone)	Antioxidant	Oral	Phase 2
					NCT01854359
		Melatonin	Antioxidant	Oral	Phase 2
					NCT01279876
	T1D	*N*-acetyl cysteine	Antioxidant	Intravenous	Phase 2
					NCT02206152
	Asthma	Alpha-lipoic acid	Antioxidant	Oral	NCT01221350
	AR				NCT00962429
	DM				NCT00187564
	DM	Glutathione	Antioxidant	Oral	NCT00858273
		Allopurinol	Antioxidant	Oral	Phase 3
					NCT02533648
	BA	Apocynin	Antioxidant	Nebulization	Phase 1
					NCT00992667
	AA	Vitamin E	Antioxidant	Oral	NCT00581048
	BTM	Silymarin (Legalon)	Antioxidant	Oral	Phase 1
					NCT01752153
	RA	Omega-3 and vitamin E	Antioxidant	Oral	Phase 1
					NCT00399282

Beyond its role as an immunomodulator, ROS can also be used to control the release of immunotherapeutics (e.g., small molecules and nucleic acids) via their encapsulation in ROS-responsive biomaterials, such as polymer-based hydrogels or nanoparticles (NPs) (*11*–*13*). This strategy helps bypass biological barriers at the tissue and subcellular levels to facilitate drug delivery to the target site (*14*, *15*). The “ROS-Responsive Biomaterials to Tune Drug Delivery for Immunotherapy” section of this review will summarize the state-of-the-art ROS-responsive platforms developed to tune the drug release kinetics for immunotherapy. Finally, the “Challenges and Considerations Toward Clinical Translation” section discusses the challenges and potential solutions for the clinical translation of ROS-modulating and ROS-responsive immunotherapy approaches and provides an outlook for future research.

## ROS-MODULATING STRATEGIES TO ENHANCE IMMUNOTHERAPIES

### ROS-modulating strategies to fight pathogens

**Fig. 1.**
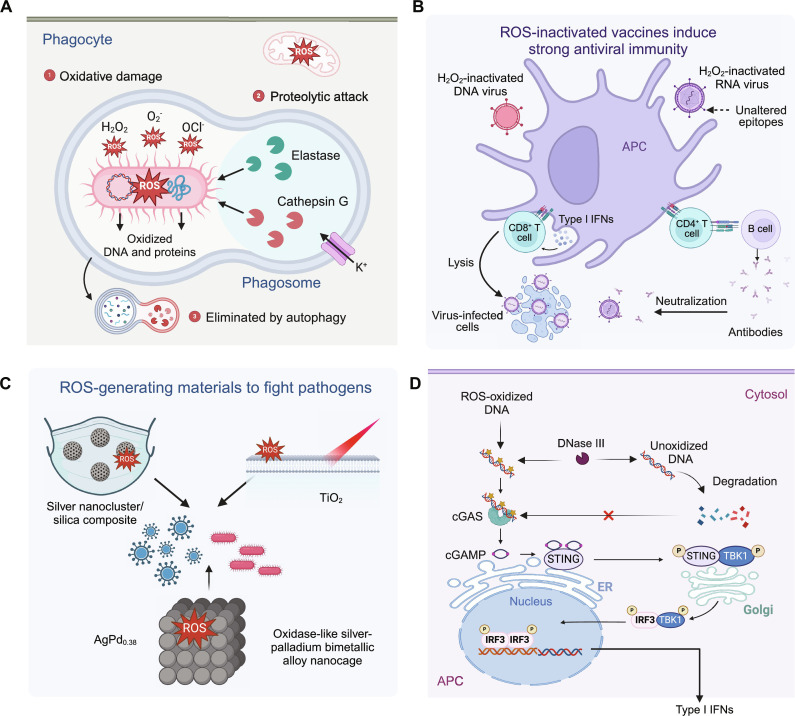
ROS-modulating strategies to fight pathogens. (**A**) Phagocytes such as macrophages and neutrophils generate ROS to kill pathogens. (**B**) Facial masks coated with ROS-generating materials (e.g., silver nanocluster/silica composite) have been designed to fight viruses, such as SARS-CoV-2. Nanozymes made of oxidase-like silver-palladium alloy nanocages generate ROS to preferentially kill bacterial over mammalian cells. NanoTechSurface (Italy) has developed a formula containing titanium dioxide and silver ions for self-disinfecting surfaces. (**C**) H_2_O_2_ has been used to inactivate viruses for the production of vaccines that can induce potent humoral and cellular immune responses. (**D**) Oxidized DNA is more resistant to degradation by DNases and therefore more efficient in activating the cGAS-STING pathway that is beneficial for promoting the induction of protective immunity. The figure was created with BioRender.

Inspired by the natural ROS-generating mechanism used by hosts to kill pathogens (*16*–*18*), biomaterials that can generate ROS have been developed to combat various pathogens (Fig. 1) (*19*–*21*). For example, facial masks containing a silver nanocluster/silica composite coating have been shown to exert viricidal effects against severe acute respiratory syndrome coronavirus 2 (SARS-CoV-2) (*22*). In addition, NanoTechSurface (Italy) has developed a formula for disinfecting surfaces via the inclusion of titanium dioxide and silver ions (*23*). Gao *et al*. (*19*) preferentially killed bacteria over mammalian cells using nanozymes made of oxidase-like silver-palladium alloy nanocages with surface-bound ROS. The optimized nanocage inhibited biofilm formation and suppressed infection in mouse models. Compared with antibiotics that often cause drug resistance upon repeated use, ROS are less likely to induce resistance and therefore represent a promising strategy for pathogen killing.

Opening the door for the use of ROS in inactivating viruses for vaccines (*24*–*26*), Amanna *et al*. (*25*) demonstrated that H_2_O_2_ rapidly inactivated RNA and DNA viruses without damaging their antigenic structures. For example, H_2_O_2_-inactivated lymphocytic choriomeningitis virus (LCMV) induced broad-spectrum virus-specific CD8^+^ T cell responses in mice, providing protection against chronic LCMV infection (*25*). Moreover, H_2_O_2_-inactivated vaccinia or West Nile virus elicited strong virus-specific neutralizing antibody responses that fully protected animals from lethal challenges. Previous studies have shown enhanced protective immunity when phagocytes increase ROS levels to kill pathogens, because the oxidative modifications to pathogen DNA induced by ROS increase both its resistance to degradation by deoxyribonuclease (DNase) III and the activation of pathogen recognition receptors (PRRs), such as STING, in the host innate immune cells (*3*, *27*). Similarly, H_2_O_2_ inactivation oxidizes the viral DNA, which has been shown to increase resistance to DNase III and activate PRRs. Thus, H_2_O_2_-inactivated viral vaccines hold potential for additional benefits in bolstering protective immunity (*28*).

### ROS-modulating strategies to fight cancer

#### Regulating ROS for cancer cell killing

**Fig. 2.**
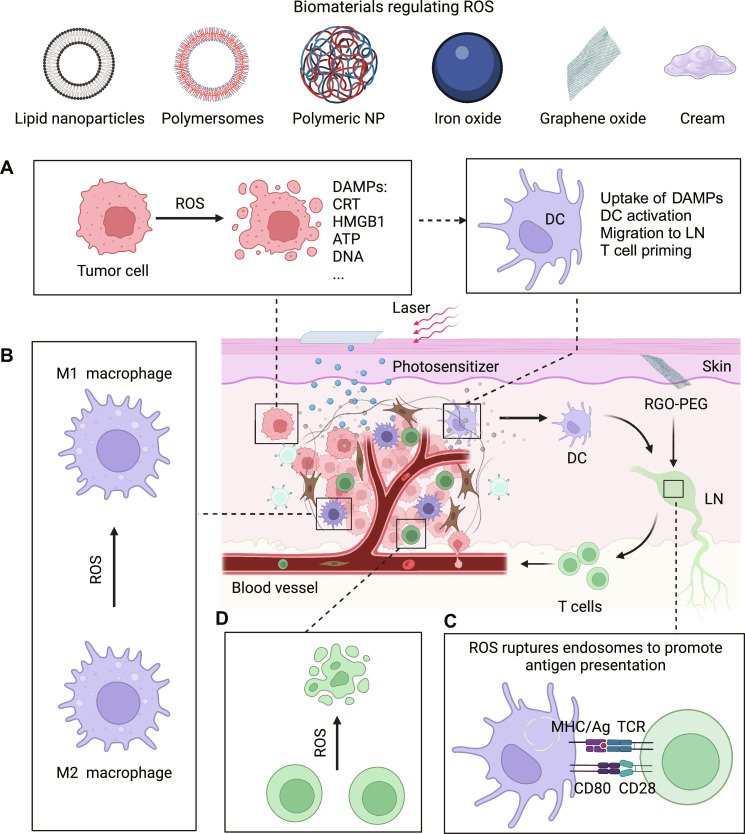
ROS-modulating strategies to fight cancer. (**A**) Free radicals and singlet oxygen can directly induce apoptosis or necrosis in cancer cells and damage tumor-related vascular tissue, which leads to further tumor cell death. Dying tumor cells can release DAMPs in oxidized forms that can shape the host immune responses in various ways and substantially affect cancer treatments. (**B**) ROS can alkalize the endosomes of DCs, leading to reduced antigen degradation and enhanced antigen presentation by the DCs. (**C**) While a basal level of endogenous ROS can polarize macrophages toward the tumor-promoting M2 phenotype, an appropriate amount of exogenously produced ROS can polarize the macrophages toward the antitumor M1 phenotype and promote immunity. (**D**) Although ROS can cause dysfunction in tumor-infiltrating T cells, the controlled generation of ROS has the potential to enhance the immunogenic cell death of tumor cells, better prime T cell responses, and ultimately improve the therapeutic outcome. The figure was created with BioRender.

One of the major goals of cancer therapy is to induce targeted cytotoxic effects in the tumor tissue without damaging normal tissues. While achieving this using traditional chemotherapies has been challenging, ROS-mediated cancer cell killing has brought new hope. This is because the dose and location of ROS can be controlled by external stimuli, such as laser (*29*, *30*), ultrasound (*31*, *32*), and ionizing radiation (*33*, *34*), making it possible to restrict the killing effect within the tumor tissue. In addition, it is believed that ROS, such as free radicals and singlet oxygen, can directly induce apoptosis or necrosis in cancer cells and damage tumor-related vascular tissue (Fig. 2), which leads to further tumor cell death (*35*).

Remarkable success in targeted cancer therapy has been achieved via photodynamic therapy (PDT), which uses photosensitizers and lasers to generate ROS. PDT has been used to treat cancer since 1978, when Dougherty *et al*. (*36*) ablated a range of cutaneous and subcutaneous tumors by intravenously injecting a photosensitizer consisting of a hematoporphyrin derivative followed by argon dye laser irradiation. Because of the limited tissue penetration capacity of light (~10 mm), PDT is mainly applied to the treatment of superficial tumors or easily accessible inner tumors, making its primary indications skin cancers (*37*, *38*). Upon topical administration, the photosensitizers aminolevulinic acid (ALA) and its derivatives methyl aminolevulinate (MAL) and hexyl aminolevulinate (HAL) can be converted to protoporphyrin IX (PPIX) via the heme synthesis pathway (*39*). Because malignant cells have enhanced uptake and reduced ferrochelatase activity compared with nonmalignant cells, more PPIX accumulates in malignant cells, which improves tumor cell killing and reduces overall toxicity. In 1990, Kennedy *et al*. (*40*) reported that the topical administration of an ALA solution to actinic keratosis or superficial basal cell carcinoma (BCC) led to a 90% complete response rate in 80 BCC lesions treated using PPIX photosensitization. Compared with ALA, its methyl ester derivative MAL is more hydrophobic and can better penetrate cells (*41*). In 2001, the European Medicines Agency (EMA) approved a 16% MAL topical cream combined with a 570- to 670-nm red light for treating actinic keratosis and BCC. In addition to skin cancers, PDT have also been used to treat other types of cancer, including breast (*42*), pancreatic (*43*), gynecologic (*44*), bladder (*45*), brain (*46*), and prostate (*47*).

To address the limited penetration of light (~10 mm), ultrasound-based sonodynamic therapy (SDT) uses sonosensitizers and ultrasound with a deeper tissue penetration ability (70 to 100 mm) to generate ROS. As such, SDT has become an attractive option for treating solid malignant tumors (*48*). The use of ultrasound allows SDT to be applied in a broader range of cancer types, such as liver, breast, brain, colon, and pancreatic cancers, as shown in many preclinical studies (*49*–*54*). Compared with PDT, SDT has not been as extensively studied in the clinical stage (*55*), although several clinical trials of SDT have been conducted since 2009. The first study combined PDT and SDT, the latter using sublingual administration of sonoflora 1 and ultrasonic irradiation (1 MHz, 2 W/cm^2^, 20 min) to treat metastatic breast cancer (*55*); two of three patients showed positive outcomes after three cycles of daily SDT treatment. In a large-scale clinical study (*56*), 115 patients with various cancers that did not respond to conventional treatments were treated with a combination of PDT and SDT, which generated ROS via a sensitizer consisting of a metallochlorin complex equivalent to sonoflora 1 and 1-MHz ultrasonic irradiation at 1 W/cm^2^. The combination treatment extended the median survival time for most of the patients. Adverse side effects typically seen in other treatments, such as bone marrow suppression and abnormal changes in hemoglobin value, white blood cell count, and platelet count, were not observed in the PDT and SDT combination therapy.

#### Regulating ROS for DC activation

DCs are antigen-presenting cells (APCs) that play critical roles in activating the adaptive immune responses, which induce further tumor regression and maintain a long-term memory of that tumor to prevent metastasis and relapse. Recent studies have shown that ROS can affect DC maturation and cross-presentation, which open up possibilities for biomaterials that can tune ROS levels in DCs to activate the immune responses. For example, Xu *et al*. (*57*) developed reduced graphene oxide (RGO) nanosheets that induced ROS generation in DCs, which alkalized endosomes and lysosomes and led to strong and sustained antigen presentation on DCs. Consequently, a single-dose vaccination induced potent T cell responses specific to the presented neoantigens and eliminated established tumors in vivo.

External stimuli can also be used to trigger ROS generation to boost antigen presentation. For example, Zhang *et al*. (*58*) conjugated the photosensitizer pheophorbide A to polyethyleneimine and then loaded the model antigen ovalbumin (OVA). This formulation responded to near-infrared light irradiation to generate ROS, which led to endosomal rupture, endosomal escape of OVA, and the consequent enhancement of OVA cross-presentation and OVA-specific CD8^+^ T cell responses. These results highlight the beneficial role of elevated ROS levels in DCs for eliciting potent T cell responses.

#### Regulating ROS for macrophage activation

The most abundant immune cells in the tumor microenvironment are tumor-associated macrophages (TAMs), accounting for up to 50% of the tumor mass (*59*, *60*). TAMs are typically divided into tumor-supportive M2 macrophages or tumoricidal M1 macrophages, where M2 TAMs are more abundant than M1 TAMs in most tumors (*61*). Previous studies have shown that the basal level of ROS can polarize macrophages to the M2 phenotype. For example, in non–small cell lung cancer (NSCLC), M2 polarization is promoted by tumoral NOX4-derived ROS through the ROS/phosphatidylinositol 3-kinase (PI3K)/Akt pathway, which contributes to tumor cell growth (*54*). Zhang *et al*. (*62*) also found that tumor-produced superoxide (O_2_·−) plays a critical role in M2 macrophage differentiation via the mitogen-activated protein kinase (MAPK) pathway. Blocking ROS via the use of butylated hydroxyanisole (BHA) suppressed differentiation in M2 but not M1 macrophages. In another study, it was found that ROS, in particular H_2_O_2_, is critical for the polarization of M2 macrophages. Inhibiting endogenous ROS production using an antioxidant decreased the expression of M2 markers, partly via Stat3 inactivation during interleukin-4 (IL-4)–induced M2 polarization (*63*). These studies indicate that basal levels of endogenous ROS may polarize the macrophages toward the tumor-supportive M2 phenotype.

Numerous studies have shown that additional production of ROS can promote polarization of macrophages toward the tumoricidal M1 phenotype. For instance, Zanganeh *et al*. (*64*) demonstrated that the iron supplement ferumoxytol induced ROS generation through Fenton reaction, and polarized the macrophages toward the M1 phenotype, as evidenced by the up-regulation of M1-related tumor necrosis factor–α (TNF-α) and CD86 and the down-regulation of M2-related CD206 and IL-10. Consequently, ferumoxytol significantly inhibited tumor growth and prevented liver metastases of adenocarcinomas. In another study, Liu *et al*. (*65*) generated ROS in tumor tissues and promoted the repolarization of M2 macrophages to M1 phenotype using zinc-PPIX grafted micelles (ZnPP PM); this M2/M1 reprogramming was blocked upon treatment with the ROS inhibitor *N*-acetyl-l-cysteine. These findings indicate that exogenous ROS can polarize macrophages toward the M1 phenotype and promote antitumor immunity. Several other studies have shown that the combination of ROS with other immunomodulators, such as imiquimod (R837), can synergistically activate macrophages and enhance their efficacy, indicating that the rational use of ROS can be a promising strategy to activate macrophages and promote subsequent adaptive antitumor immunity (*50*).

#### Regulating ROS for T cell activation

ROS generation and scavenging are carefully controlled processes, as imbalances could affect T cell immunity. Along these lines, recent studies have suggested that dysfunctional T cells have high levels of mitochondrial ROS (mtROS) (*66*). For example, Kaminski *et al*. (*67*) showed that ROS derived from the mitochondria of T cells can induce Fas ligand (FasL or CD95L) expression, which mediated the activation-induced cell death (AICD) of T cells; this process was dependent on O_2_^−^ instead of H_2_O_2_ (*68*). Scharping *et al*. (*69*) found that elevated mtROS induced by continuous stimulation led to T cell exhaustion. Vardhana *et al*. (*70*) also found that chronic antigen stimulation increased mtROS accumulation in T cells, which impeded their proliferation and self-renewal. As this T cell suppression/exhaustion could be mitigated by decreasing cellular ROS using antioxidants such as *N*-acetyl-l-cysteine, scavenging the excess mtROS provides a potential approach for regulating T cell immunity. To neutralize ROS, Shi *et al*. (*71*) recently developed T cell–targeting fusogenic liposomes equipped with 2,2,6,6-tetramethylpiperidine (TEMP), which protected T cells from oxidation-induced activity loss and efficiently inhibited tumor growth in multiple mouse tumor models.

The priming of T cell immunity can be substantially affected by a combination of ROS modulators and chemotherapeutics, which can affect the fate of tumor cells in the tumor microenvironment. Recent mechanistic studies have shown that chemotherapeutics can induce immunogenic tumor cell death (ICD). During this process, tumor cells can up-regulate “eat me” signals like calreticulin (CRT) that results in the secretion of a variety of DAMPs, such as HMGB1 (*72*–*74*). Furthermore, the DC-mediated phagocytosis of tumor cells undergoing immunogenic cell death further activates antitumor T cells (*75*–*77*). Because elevated ROS levels can oxidize HMGB1 and compromise its immunostimulatory activity, strategies for reducing ROS levels are promising for improving T cell responses (*78*). To clear ROS, Deng *et al*. (*78*) developed a ROS nanoscavenger containing a cleavable poly(ethylene glycol) (PEG) corona that shielded a ligand targeting the extracellular matrix (ECM). Upon reaching the tumor microenvironment, the acidic pH cleaved the PEG corona to expose the ECM-targeting peptide, thus allowing the nanoscavenger to anchor on the ECM and scavenge ROS in a continuous manner. Furthermore, ROS oxidation disrupted the structure of the nanoscavenger, which released the drug oleandrin to induce the immunogenic cell death of tumor cells. Because of the absence of ROS, dying tumor cells were able to provide antigens and nonoxidized HMGB1 to prime and promote the function of T cells for effective anticancer therapy.

Strategies that boosted ROS generation to kill cancers also enhanced the T cell responses. For example, He *et al*. (*79*) developed nanoscale coordination polymer (NCP) core-shell NPs containing the chemotherapy oxaliplatin and the photosensitizer pyrolipid. Laser irradiation then triggered the release of cytotoxic ROS, which synergized with the oxaliplatin and led to tumor cell death and induced immunogenic cell death, as shown by the exposure of CRT on the surface of tumor cells. Consequently, this treatment generated tumor antigen–specific T cell responses and eliminated established tumors in combination with a checkpoint blockade. In another study, Duan *et al*. (*80*) developed NCP core-shell particles containing oxaliplatin and ROS-inducing dihydroartemisinin, which synergized to kill tumor cells and induce immunogenic cell death. This cell death led to the expression of CRT, the release of HMBG1, and potent T cell responses. While excess ROS can adversely affect T cells, these results regardless indicate that ROS-mediated tumor cell killing in conjunction with other therapies can improve the induction of immunogenic cancer cell death to prime T cell responses.

### ROS-modulating biomaterials to fight autoimmune and inflammatory diseases

#### Regulating ROS for the treatment of inflammatory bowel diseases

**Fig. 3.**
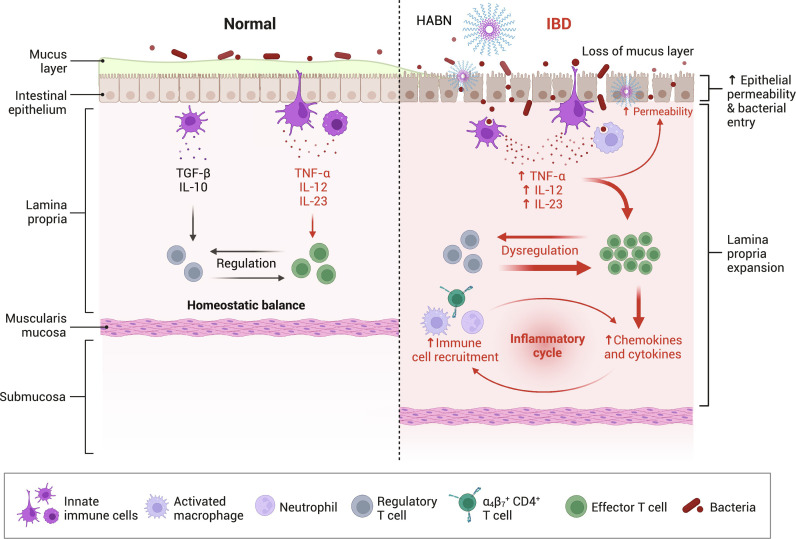
ROS-modulating strategies to fight autoimmune diseases. Overproduction of ROS in the gastrointestinal tract can amplify inflammatory responses, leading to intestinal endothelial cell damage and dysbiosis of the gut microbiota. ROS-scavenging materials such as bilirubin–hyaluronic acid nanoparticles (HABN) can reduce apoptosis in colonic epithelial cells and increase the overall richness and diversity of microorganisms, such as *A. muciniphila* and *Clostridium* XIVα, that play essential roles in gut homeostasis, resulting in improved therapeutic efficacy against colitis. The figure was created with BioRender. TGF-β, transforming growth factor–β.

Traditional treatments for autoimmune diseases, such as inflammatory bowel diseases (IBDs), suppress immune responses (*81*). However, this can cause systemic side effects while failing to address the underlying causes of IBD, such as damage to the intestinal barrier functions and abnormal changes to gut commensal microorganisms (*82*). Recent studies have shown that the overproduction of ROS in the gastrointestinal tract can amplify inflammatory responses, leading to intestinal endothelial cell damage and dysbiosis of the gut microbiota (*83*–*85*). These findings have inspired the use of ROS scavengers to treat IBD (*86*–*88*) by alleviating the symptoms while also better targeting the causes, such as the dysbiosis of the gut commensal microorganisms (Fig. 3).

In one study using this technique to treat IBD, Lee *et al*. (*89*) conjugated the ROS scavenger bilirubin (BR) to hyaluronic acid (HA) to counter its hydrophobicity and toxicity. Although BR is poorly soluble in water, these conjugates spontaneously formed homogeneous nanoparticles (HABNs) in the aqueous phase and still exhibited potent ROS-scavenging capabilities. Orally administered HABN efficiently accumulated in the inflamed intestinal epithelium and proinflammatory macrophages through HA-CD44 interactions. Furthermore, HABN reduced the apoptosis of colonic epithelial cells in a murine model of dextran sodium sulfate–induced acute colitis. Remarkably, HABN increased the overall richness and diversity of microorganisms that play essential roles in gut homeostasis, such as *Akkermansia muciniphila* and *Clostridium* XIVα, resulting in potent therapeutic efficacy against colitis.

The therapeutic efficacy of ROS scavengers toward autoimmune diseases can be further improved through their combined use with other modalities. For example, Liu *et al*. (*90*) created another IBD therapy that involved a highly hydrophobic ROS scavenger, poly(propylene sulfide) (PPS), conjugated to HA to generate homogeneous self-assembled nanoparticles (HPNs). HPN maintained the potent ROS-scavenging activity of PPS. To achieve target delivery to the disease site, the authors conjugated HPN to probiotics, known to colonize the colon, that were precoated with a poly-norepinephrine (NE) film with strong adhesive properties. Consequently, this platform also exhibited potent therapeutic efficacy on a murine dextran sulfate sodium (DSS)–induced acute colitis model.

#### Regulating ROS for the treatment of rheumatoid arthritis

Rheumatoid arthritis (RA) is an autoimmune disease with inflammatory synovitis and joint disability (*91*, *92*). Although the pathogenesis of RA has not been clearly elucidated, the mechanism involves unwanted immune responses that attack the joints. It has been shown that ROS accumulation and mitochondrial damage in RA joints not only affect the metabolic processes of immune cells and pathological changes in fibroblast synoviocytes but also up-regulate multiple inflammatory pathways, which ultimately lead to the progression of inflammation (*93*, *94*). Additionally, ROS and mitochondrial damage are involved in angiogenesis and bone destruction, thereby accelerating the progression of RA (*93*). These findings have promoted the exploration of ROS scavengers as a potential treatment for RA (*95*–*99*).

In one recent study, Zhou *et al*. (*99*) developed a ROS-responsive micelle encapsulating a Toll-like receptor 4 (TLR4) inhibitor rhein (RH) and superoxide eliminator CeO*_X_* to alleviate aberrant inflammatory responses and oxidative stress in synovial tissues. To prepare the ROS-responsive micelles, the hydrophobic thioketal-bridged adamantine-RH complex was anchored to the hydrophilic HA–β-cyclodextrin complex via the host-guest interaction of adamantine and β-cyclodextrin, followed by coordination of CeO*_X_* onto the carboxyl group of HA to obtain HA@RH-CeO*_X_* micelles (*99*). Upon intra-articular injection, the HA@RH-CeO*_X_* micelles were selectively engulfed by M1 macrophages. The thioketal linkage was cleaved under the high cellular ROS levels, which triggered the disintegration of the micellar structure, leading to release of RH and CeO*_X_* into cytosol. The cellular ROS was further decomposed by Ce^3+^/Ce^4+^ redox pair via its superoxide dismutase (SOD)–like enzymatic activity, effectively alleviating the oxidative stress in M1 macrophages and improving the articular functions (*99*).

Because of the overexpression of inflammatory cytokines in joints, the combination of ROS scavengers and small interfering RNA (siRNA) knocking down inflammatory cytokines may improve the therapeutic efficacy for RA. For example, Shang *et al*. (*97*) developed a biomimetic nanocomplex encapsulating TNF-α siRNA and catalase coated with the macrophage membrane. After systemic administration, the nanocomplex exhibited long circulation time and effective accumulation in joints. Catalase in the nanocomplex catalyzed the decomposition of H_2_O_2_ into O_2_ in the oxidative microenvironment of the inflamed joints. The inner positively charged nanocomplex core was exposed simultaneously to facilitate internalization into synovial macrophages, leading to efficient TNF-α silencing (*97*). All the studies above indicate that modulating ROS is a promising strategy for the treatment of RA.

#### Regulating ROS for the treatment of asthma

Asthma is the second most prevalent chronic lung disease in the world (*100*). Increasing evidence has shown that neutrophil extracellular traps (NETs), which are large, extracellular, web-like structures primarily composed of DNA from neutrophils, influence the underlying cardinal features of allergic asthma (*101*). The dysregulated formation of NETs is a main cause of many inflammatory diseases. Previous studies have shown that ROS, produced by NADPH (reduced form of nicotinamide adenine dinucleotide phosphate) oxidase or mitochondria upon activation of innate immune receptors, can activate myeloperoxidase, neutrophil elastase, and protein-arginine deiminase type 4 to promote chromatin decondensation, leading to the formation of NETs (*101*). Thus, scavenging ROS represents a promising strategy to inhibit NETs for asthma treatment.

Toward this goal, Li *et al*. (*102*, *103*) developed a ROS-scavenging NP by covalently conjugating Tempol (an SOD analog that neutralizes ROS) and 4-(hydroxymethyl) phenylboronic acid pinacol ester (an H_2_O_2_-eliminating material) onto β-cyclodextrin. These NPs efficiently accumulated in lung neutrophils of asthmatic mice following intravenous injection or inhalation, thereby substantially mitigating oxidative stress, suppressing inflammatory responses, and improving pulmonary functions in mice with allergic/neutrophilic asthma (*102*). Altogether, the above results indicate that ROS-scavenging materials hold great potential to treat asthma.

## ROS-RESPONSIVE BIOMATERIALS TO TUNE DRUG DELIVERY FOR IMMUNOTHERAPY

### ROS-responsive drug release to trigger in situ ICD

**Fig. 4.**
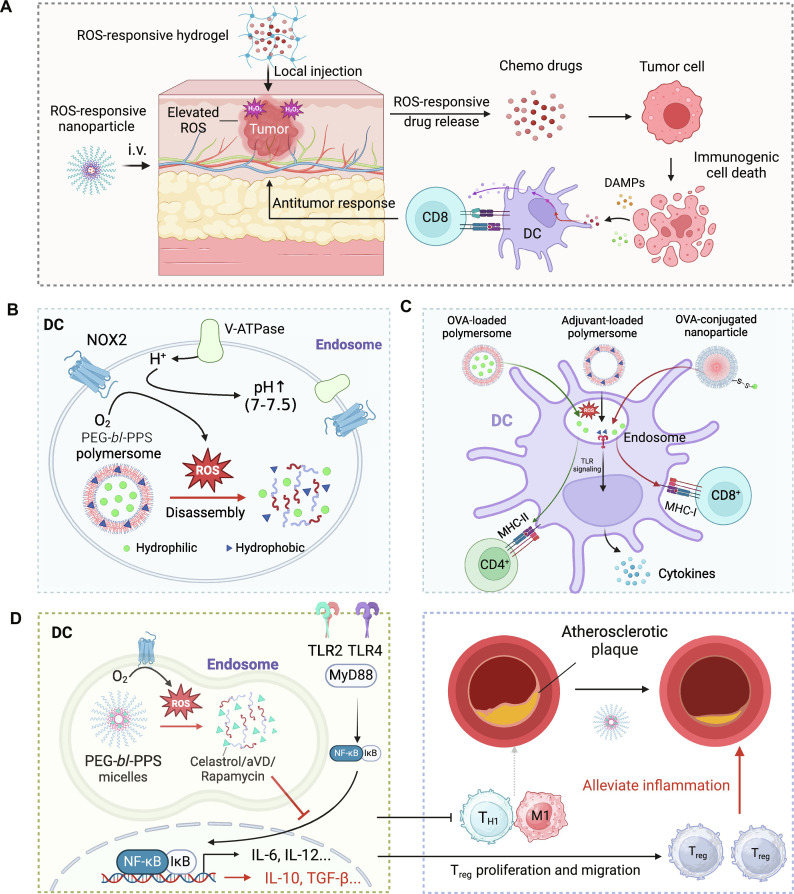
ROS-responsive biomaterials to tune drug release for immunotherapy. (**A**) ROS-responsive hydrogels or NPs release anticancer drugs in the presence of elevated ROS levels in the tumor microenvironment, leading to immunogenic cancer cell death and activation of antitumor T cell responses. (**B**) ROS-responsive PEG-*bl*-PPS PSs release drugs in the endosomes of DC, which have elevated ROS levels, thus paving the way to use PEG-*bl*-PPS for delivering different immunotherapeutics and tuning the functions of DC. (**C**) The targeted release of adjuvant (e.g., R848) from PSs resulted in enhanced DC maturation and induction of proinflammatory cytokines such as IL-6 and IL-12. Moreover, antigen loaded into PEG-*bl*-PPS-PSs is delivered more efficiently into the major histocompatibility complex class II (MHC-II) pathway than the MHC-I pathway, inducing potent CD4^+^ T cell responses upon combination with the adjuvant CpG. In contrast, antigen conjugated on the surfaces of PEG-*bl*-PPS-NPs is delivered more efficiently into the MHC-I pathway, inducing potent CD8^+^ T cell responses upon combination with CpG. (**D**) PEG-*bl*-PPS micelles release anti-inflammatory drugs in ROS-rich endosomes of DC, leading to immunosuppression in the atherosclerotic plaque and reduced plaque lesions. The figure was created with BioRender.

The therapeutic induction of ICD requires efficient drug release in tumor tissue but not normal tissue to maximize the efficacy while minimizing off-target effects (*104*). Because of the elevated levels of ROS in the tumor microenvironment compared with normal tissues (*105*), ROS can be used to specifically trigger drug release in the tumor while avoiding toxicity in normal tissues (Fig. 4A).

As a proof of concept of this targeted release, Wang *et al*. (*11*) developed a ROS-responsive hydrogel made of poly(vinyl alcohol) with the ROS-labile linker *N*^1^-(4-boronobenzyl)-*N*^3^-(4-boronophenyl)-*N*^1^,*N*^1^,*N*^3^,*N*^3^-tetramethylpropane-1,3-diaminium. The hydrogel was loaded with the chemotherapeutic gemcitabine for cancer therapy (*11*). Upon implantation to the tumor site, the abundant ROS triggered the release of the gemcitabine from the hydrogel. The released chemotherapeutics induced immunogenic cancer cell death and triggered antitumor T cell responses that synergized with anti–PD-L1 antibody treatment to address poorly immunogenic tumors, such as B16F10 and 4T1.

In addition to local administration of ROS-responsive materials, systemic delivery of NPs containing ROS-responsive linkers (Table 2), such as arylboronic ester, thioketal, disulfide, and diselenide groups, has also been used to release drugs in response to elevated ROS levels (*106*). For example, to selectively release doxycycline (DOX) in tumors with elevated ROS levels, Deepagan *et al*. (*107*) developed diselenide-crosslinked micelles (DCMs). The shell of DCMs was fabricated from selenol-containing triblock copolymers, with DOX encapsulated inside the hydrophobic core. These DCMs remained stable for at least 6 days in normal conditions (20 nM H_2_O_2_), although they displayed a drastic release (65%) of DOX upon exposure to 100 mM H_2_O_2_ (which approximates the intratumoral H_2_O_2_ concentration). Furthermore, after systemic administration, significantly more DOX could be delivered to the tumor region using DOX-DCMs as compared to free DOX and noncrosslinked DOX micelles. As expected, the DOX-DCM–treated group exhibited better antitumor efficacy than the other two formulations, indicating that ROS-triggered drug release in the tumor region is beneficial for the therapeutic effect.

**Table 2.** ROS-responsive materials for drug release and delivery. DOX, doxorubicin; PEG, polyethylene glycol; PPS, propylene sulfide; DMA, *N*,*N*-dimethylacrylamide; PTX, paclitaxel; PBE, phenyl boronic ester; TPGS, d-α-tocopheryl polyethylene glycol succinate; PS, phenyl sulfide; AA, aminoacrylate.

Mechanism for drug release	ROS-responsive linkers	Nanoplatforms for drug delivery	Applications	References
Carrier disassembly triggered by ROS-induced material solubility change	Thioether	DOX-loaded polymeric micelle	NCI-H460 tumor model	(*161*)
	Sulfide	PEG-*bl*-PPS block copolymer filomicelles	Atherosclerotic mice; human plasmacytoid dendritic cells (pDCs)	(*118*, *120*, *162*)
		PEG-*bl*-PPS polymeric bicontinuous nanospheres	RAW 264.7 cells MCF7 breast cancer cells	(*137*)
		poly(PS_74_-b-DMA_310_) micelles	RAW 264.7 cells	(*163*)
		DOX-loaded phenyl sulfide–containing mesoporous silicon	MCF-7 breast cancer cells	(*164*)
	Monoselenide	Monoselenide-containing amphiphilic hyperbranched copolymer micelle	HeLa cells	(*165*)
	Ferrolene	PTX-loaded ferrocene-containing amphiphilic block copolymers	A549 lung cancer cells	(*166*)
	Phenyl boronic acid	DNA-loaded charge-reversal lipidic polyplex	A549 lung tumor model	(*167*)
	Phenyl boronic ester	PBE-containing siRNA-loaded polymeric nanomedicine	U87MG glioma model	(*168*)
Carrier degradation caused by ROS-responsive linker cleavage	Thioester	PTX-loaded TPGS-poly(β-thioester) nanoparticles	SCC-7 tumor model	(*169*)
	Diselenide	DOX-loaded diselenide-crosslinked micelles	PC3 tumor model	(*107*)
	Tellurium	Cisplatin and indocyanine green–loaded tellurium-containing polymer	MDA-MB-231 tumor model	(*170*)
	Oxalate ester	Palmitoyl ascorbate hybrid micelles	4T1 tumor model	(*171*)
	Oligoproline	PEGylated oligoproline-containing polymeric nanocarriers	Vascular smooth muscle cells (VSMCs)	(*172*)
	Aminoacrylate	AA-containing PS- oligoethylenimine (OEI)–conjugated polymer	HeLa cells	(*173*)
ROS-responsive carrier-drug linker cleavage	Thioketal	Thioketal-linked polyphosphoester-DOX conjugate	MDA-MB-231 tumor model	(*174*)

### ROS-responsive drug release to regulate immune function of APCs

#### The biochemical linkage between ROS generation and ROS-modulated drug release in APCs

Phagocytes, including neutrophils, macrophages, and DCs, can engulf exogenous pathogens or dead cells to prevent infectious diseases and maintain homeostasis (*17*). Although multiple types of cells have antigen-presenting capacity in vitro, cumulative evidence has shown that DC is the main protagonist in antigen presentation and T cell priming in vivo (*108*). The robust antigen-presenting ability of DCs is attributed to their specialized pH and redox states within the early endocytic compartment (*109*). After internalizing the exogenous antigens in DCs, NADPH oxidases (NOX2) are recruited to the endosomal membrane to generate ROS from oxygens. The endosome milieu then undergoes a mild alkalinization due to the consumption of protons, which leads to the elevation of endosomal pH (*110*, *111*). The alleviated acidity and low level of proteolytic enzymatic activity allow gentle degradation and efficient export of antigens, thus enhancing the antigen presentation to the CD8^+^ T cells (*17*). However, in other types of APCs, like macrophages and neutrophils, the rapid acidification of early endosomes after antigen internalization and much higher proteolytic enzymatic activity in late endosomes substantially impair their antigen-presenting efficacy (*112*, *113*).

On the basis of the subtle and durable ROS generation in early endosomes of DCs, Scott *et al*. (*114*) developed a ROS-responsive nanodrug delivery system that can specifically release drugs in the DC endosome and regulate the immune functions of DCs. The ROS-responsive property of this nanocarrier relies on a polymeric component called PPS, which is highly hydrophobic in normal conditions and undergoes a two-step oxidative conversion from hydrophobic PPS to less hydrophobic poly(propylene sulfoxide) and eventually the highly hydrophilic poly(propylene sulfone) (*115*). A nanoscale polymersome (PS) formed by the block copolymer consisting of hydrophilic PEG and hydrophobic PPS (called PEG-*bl*-PPS PS) allows the stable encapsulation of both hydrophobic drug molecules within vesicle membrane and hydrophilic molecules within vesicle interior (*114*). Upon exposure to an oxidative environment within DC endosomes, PEG-*bl*-PPS PS undergoes a rapid carrier disassembly followed by efficient payload release, thus paving the way to use PEG-*bl*-PPS for delivering different immunotherapeutics and tuning the functions of DC (Fig. 4B).

#### Application of ROS-responsive PEG-PPS biomaterials in immune activation

PEG-*bl*-PPS PSs have been used to deliver immunostimulatory molecules like immune adjuvants and antigens to the endosomes of DCs, inducing potent immune responses (*114*, *116*). For instance, Scott *et al*. (*114*) encapsulated a model antigen OVA and TLR7/8 agonist R848 or TLR7 agonist gardiquimod in PEG-*bl*-PPS PSs. Those drug-loaded PSs exhibited effective payload release under oxidative conditions like H_2_O_2_ solutions and ROS-rich DC endosomes. The specific release of OVA protein in DCs boosted antigen cross-presentation and priming of CD8^+^ T cells in vitro. The targeted release of R848 and gardiquimod resulted in enhanced DC maturation and induction of proinflammatory cytokines such as IL-6 and IL-12 (*114*). A subsequent study by Stano *et al*. (*116*) compared the intensities and types of immune responses elicited by watery-core PEG-*bl*-PPS PSs and solid-core PEG-stabilized PPS NPs. Both PSs and NPs displayed ROS-responsive drug release, but PSs had antigens encapsulated inside and NPs had antigens displayed on the surface. The results showed that PSs tended to induce CD4^+^ T cell responses, while NPs tended to induce CD8^+^ T cell responses (Fig. 4C), indicating that although the DC-specific nanovaccines based on PEG-*bl*-PPS materials can induce strong T cell responses, it is important to optimize the antigen loading methods to elicit both strong CD4^+^ and CD8^+^ T cell responses (*116*). The above examples demonstrate the efficacy of ROS-sensitive PEG-*bl*-PPS–based nanovaccines in immune activation and show great potential in therapeutic applications. Further investigations are needed to explore their clinical use in combating diseases such as infectious diseases and cancers.

#### Application of ROS-responsive PEG-PPS biomaterials in immunosuppression

Beyond delivering immunostimulatory drugs to activate DCs, the ROS-responsive PEG-*bl*-PPS platform is also applicable for delivering immunosuppressive agents to DCs to treat autoimmune diseases (*117*–*120*). For instance, atherosclerosis, one of the main causes of cardiovascular disease, is closely related to chronic inflammation (*121*). Excessive oxidized low-density lipoprotein (LDL) in atherosclerosis patients binds to pattern recognition receptors such as TLR2 and TLR4 to activate downstream nuclear factor κB (NF-κB) signaling, which promotes secretion of a series of proinflammatory cytokines to induce cell death and worsen oxidative stress (*122*). This process triggers positive feedback of proinflammatory signaling in vascular lesions and eventually exacerbates atherosclerosis (*121*). Small-molecule NF-κB inhibitors such as celastrol and 1,25-dihydroxyvitamin D3 (aVD) can directly suppress NF-κB–mediated inflammation and hold great potential in treating atherosclerosis (*123*, *124*). However, direct administration (oral or intraperitoneal) of these small molecules suffers from limited therapeutic efficacy and severe systemic side effects due to their broad biodistribution, poor stability, low bioavailability, and variable pharmacological targets (*117*, *125*, *126*). To address these challenges, Scott and his coworkers (*117*, *118*) used the PEG-*bl*-PPS micelles to deliver the celastrol and aVD safely and effectively to immune cells and specifically release those anti-inflammatory agents in DC endosomes in response to the elevated ROS levels. This intravenously injectable nanoplatform not only improved the systemic safety of celastrol and aVD but also significantly reduced the dose needed to induce anti-inflammatory responses and eventually reduced the plaque area in atherosclerotic lesions (Fig. 4D). Similarly, subcutaneously injected PEG-*bl*-PPS micelles were also able to alleviate graft-versus-host disease (GVHD) during islet transplantation by overcoming the pharmacokinetic issues and severe side effects of immunosuppressive rapamycin (*119*).

These studies on PEG-*bl*-PPS–based nanoplatform inspire a comprehensive understanding of the unique biochemical characteristics of target cell populations, especially in complex diseases such as immuno-related, cancerous, and metabolic disorders, to achieve targeted biological regulation through stimuli-responsive delivery systems.

### ROS-mediated targeted delivery

**Fig. 5.**
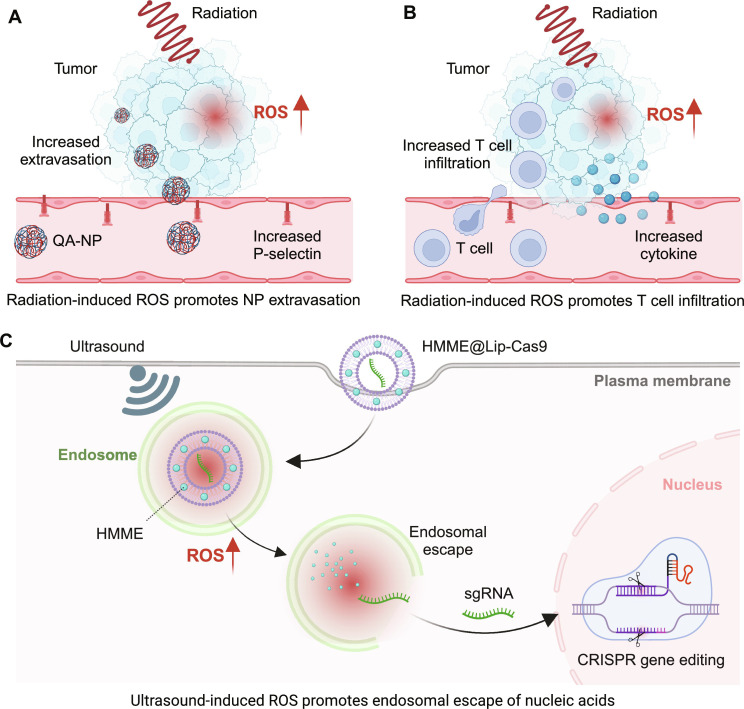
ROS-responsive biomaterials for tuning drug delivery. (**A**) External stimuli-induced ROS can up-regulate P-selectin expression on endothelial cells within the tumor tissue, which promotes the accumulation of NPs bearing the P-selectin targeting ligand (QA). (**B**) ROS-induced inflammation in the tumor can also be used to promote the infiltration of adoptively transferred T cells, which are considered live therapeutics. (**C**) At the subcellular level, ROS can enhance the permeability of endosomes, thus promoting the endosomal escape of nucleic acids. The figure was created with BioRender.

To interact with the target and induce the desired pharmacological effect, therapeutics typically need to extravasate from the blood, enter the target cells, and escape from the endosomes. While it is essential to engineer therapeutics with appropriate targeting moieties to enhance targeted delivery (*127*), modulating the microenvironment in the target tissue could also facilitate the enrichment of engineered therapeutics in the correct location. In this regard, ROS can be used to tune the microenvironment at the tissue and subcellular levels to ultimately promote drug delivery to the target site (Fig. 5).

For example, the expression of P-selectin on endothelial cells can facilitate the binding and retention of NPs and improve their extravasation to the disease site (e.g., tumor) (*127*, *128*), but this expression can be relatively low around target sites. Nevertheless, ROS has been shown to induce inflammation and up-regulate the expression of P-selectin on endothelial cells (*129*, *130*), making it possible to use ROS to promote drug delivery to the target site. This up-regulation can be inhibited by exogenous antioxidants, indicating that it is highly dependent on ROS. To test the feasibility of this approach, Xu *et al*. (*15*) developed an NP decorated with quinic acid (QA), which can recognize P-selectin on endothelial cells. Compared with nonmodified NPs, QA-NP exhibited enhanced accumulation in the tumor due to the basal level of P-selectin expression around the peritumoral blood vessels. Remarkably, irradiation over the tumor region further up-regulated P-selectin (*130*) on the peritumoral vessel and promoted the additional accumulation of QA-NP in the tumor region. QA-NP loaded with the chemotherapeutic paclitaxel (PTX) significantly enhanced the therapeutic efficacy on multiple tumor models over PTX-loaded NP without the QA modification. Because ROS are known to directly up-regulate P-selectin, the controlled generation of ROS using external stimuli at the target site can be a feasible approach for tuning the drug delivery profile.

In addition to improving NP delivery, the inflammatory microenvironment induced by irradiation can be used to tune the delivery of adoptively transferred T cells, which are emerging as “live therapeutics” that require efficient tumor infiltration to kill tumor cells (*131*). Ganss *et al*. (*132*) demonstrated that irradiation over the target tumor site followed by the adoptive transfer of T cells led to complete tumor regression. In contrast, tumor-specific T cells alone failed to eradicate solid tumors. The enhanced therapeutic outcome of the combination therapy is partially due to the proinflammatory environment facilitating the intratumoral infiltration of T cells.

After reaching the target tissue, nanomedicines are typically phagocytosed into endosomes, despite that the targets of many drugs are located outside the endosomes (e.g., cytosol or nucleus) (*133*–*135*). Thus, it is essential to release drugs from the endosomes. ROS has been shown to promote the endosomal escape of selected drugs. For instance, to achieve precise and effective gene editing in tumor sites, Yin *et al*. (*49*) developed an ultrasound-responsive liposomal CRISPR-Cas9 that contains the sonosensitizer hematoporphyrin monomethyl ether (HMME) (HMME@Lip-Cas9). In their study, the authors encapsulated HMME along with the Cas9/single-guide RNA (sgRNA) ribonucleoprotein targeting nuclear factor erythroid 2-related factor 2 (NFE2L2; a protein that can compromise the effect of SDT) in liposomes and simultaneously delivered them to the tumor site. Ultrasonic irradiation on the HMME-containing tumor tissue generated a large amount of ROS, which caused oxidative damage to the cell that disrupted the endosomal membrane, boosted the endosomal escape of Cas9/sgRNA ribonucleoprotein, and efficiently promoted its nuclear transportation and the subsequent deletion of NFE2L2. Consequently, the HMME@Lip-Cas9 platform augmented the therapeutic efficacy of SDT on hepatocellular carcinoma, indicating that ROS can be a valuable tool to boost cytosolic drug delivery.

## CHALLENGES AND CONSIDERATIONS TOWARD CLINICAL TRANSLATION

### Achieving spatiotemporal control of ROS

To maximize therapeutic effects, ROS levels must be precisely modulated in the diseased regions. However, ROS inducers or scavengers typically cannot efficiently reach the target site due to multiple levels of physiological barriers. Nanotechnology holds great potential for addressing this issue, as nanoscale formulations have demonstrated improved pharmacokinetics and enhanced delivery profiles of these compounds following local or systemic delivery to the target site. The performance of nanoscale formulations can be further enhanced by tuning the size, charge, shape, and surface properties (*136*).

The spatiotemporal control of ROS generation from photosensitizers and sonosensitizers can be achieved through external stimuli, such as laser, ultrasound, or radiation. However, the existing pool of sensitizers are limited and have poor safety, low stability, and low efficiency for ROS generation. Therefore, developing novel sensitizers with reduced cytotoxicity, improved drug stability, and high quantum yield is of great importance. For example, a lot of efforts have been devoted to developing innovative photosensitizers. The recent strategies were presented in great detail in another review article (*30*).

Broad biodistribution and inefficient targeted delivery of free small-molecule sensitizers can limit their therapeutic efficacy in vivo. Therefore, developing a stable and targeted delivery platform for the sensitizers with on-demand drug release and off-on cytotoxicity is critical. Modak *et al*. (*137*) developed bicontinuous nanospheres (BCNs) prepared from oxidation-sensitive biomaterial PEG-*bl*-PPS loaded with the photosensitizer pheophorbide A and chemotherapeutic camptothecin. This condensed nanostructure had a larger amount of internal PPS and displayed a ROS-scavenging property that maintained the carrier stability under endogenous oxidation conditions and protected cells from cytotoxic effect of pheophorbide A and camptothecin. Upon exposure to photoirradiation, the nanostructure underwent a morphological shift from larger BCNs to micelles and rapidly released pheophorbide A and camptothecin from BCNs, leading to the on-demand cytosolic delivery and off-on cytotoxicity of the proapoptotic drugs (*137*). This nanocarrier might be used in PDTs to combat cancers after fully validating its safety and efficacy in vivo.

Moreover, each stimulus that is used to trigger ROS generation has its own limitations. For example, lasers have a short penetration depth and are therefore not suitable for deep tissues; ultrasound has a deeper penetration depth, but it may be less effective for tissues containing air bubbles or strong reflective properties, such as bones. Consequently, the method used for ROS generation should be carefully chosen based on the context and specific requirements.

ROS generation naturally requires oxygen, but the disease site is typically hypoxic, compromising ROS generation efficiency. Supplementing exogenous oxygen at the disease site is one potential strategy for combating this issue. While this approach has shown some improvement in terms of ROS generation (*138*), the increased complexity may prevent its wider use. Another method would be to develop sensitizers that can generate ROS even without oxygen. For example, Yao *et al*. (*139*) developed a novel photosensitizer for PDT therapy, which was able to kill tumor cells independent of oxygen, indicating that the strategy was feasible in the context of cancer therapy.

### Gaining deeper understanding of the mechanism of action for ROS

The ability of ROS-modulating materials to directly induce biological effects has made them useful across broad applications. However, it is essential to recognize that ROS includes multiple species, including peroxides, superoxide, hydroxyl radicals, singlet oxygen, and α-oxygen, and it is not straightforward to determine which of these species are responsible for mediating the desired outcome. Therefore, further studies that can precisely measure the levels of different ROS species and uncover their individual impact on the biological effect, spanning different cell types, are of great importance.

To date, there have been a number of commercially available probes such as dihydroethidium (DHE) (*140*), dihydrorhodamine (DHR) (*141*), and dichlorodihydrofluorescein diacetate (DCFH-DA) (*142*) for ROS detection. However, most of these traditional ROS probes display unsatisfied specificity, sensitivity, and accuracy, and cannot distinguish different types of ROS (*143*–*145*). Although some specific ROS probes have been developed in recent years, the targeted types of ROS are focused on H_2_O_2_ (*146*, *147*) and O_2_·− (*148*). Therefore, it is important to expand the toolbox of specific ROS probes targeting other types of ROS.

Apart from small molecule–based probes for ROS detection in vitro, biomaterials that allow ROS quantification in vivo are of great significance. This can be achieved by biomaterials with ROS-activatable fluorescence change. For example, Du *et al*. (*149*) synthesized the fluorescent dye perylene bisimide (PBI)-bridged tetrablock copolymer (PEG-PPS-PBI-PPS-PEG), which can form PSs in phosphate-buffered saline. Free PBI emitted green fluorescence (550 nm), but its fluorescence underwent a red shift to 640 nm during the self-assembly of PSs. In the presence of elevated levels of ROS, the tetrablock copolymer disassembled into PEG-PPS diblock copolymer and released free PBI, shifting the fluorescence from 640 nm to 550 nm. After subcutaneously injecting the PSs in mice, the remarkable fluorescence shift from 640 to 550 nm was detected in lymphatic DCs and macrophages, indicating that the tetrablock copolymer was degraded within endosomes of DCs and macrophages due to their excessive levels of ROS. Besides, the discrepancy of spectrum shift among DCs, macrophages, and other immune cells further demonstrated their distinctive roles in modulating the immune responses.

To identify the individual role of different ROS species in biological activities, it is necessary to deplete certain types of ROS specifically and efficiently. So far, only pan-antioxidants such as *N*-acetylcysteine (NAC) (*150*), glutathione (GSH) (*151*), vitamin C (*152*), and vitamin E (*153*) can act as broad-spectrum ROS scavengers, which fail to block specific ROS species. Therefore, scavengers targeting specific types of ROS are urgently needed to uncover the impact of individual ROS on multiple biological processes or on the ROS-modulated drug delivery, which is a challenging but very interesting research direction in this area.

Another critical issue to consider is the timing, dose, and location of ROS, all of which could affect the outcomes. For example, while transiently elevating ROS levels may enhance tumor cell killing, the extended elevation of ROS may compromise the activation and function of antitumor T cells (*154*). Therefore, it is of great importance to fully understand the biological effects of ROS on different cell types before using ROS for the treatment of diseases. This will help guide the delivery of the right amount of ROS at the right time and location to precisely regulate cellular functions. To achieve such fine regulation of ROS, it is necessary to design drug delivery vehicles with high tissue- or cell-targeting ability, as well as combining with exogenous stimuli that can precisely tune the intensity and duration of ROS production.

### Enhancing the breadth and capability of ROS-responsive biomaterials

Recent studies have shown that ROS-triggered drug release is a promising strategy for delivering the required amount of drug at the desired place and time. While existing biomaterials have demonstrated potential of drug release in response to elevated ROS levels, their current scope remains limited and may not meet the therapeutic need. The performance of ROS-responsive biomaterials may introduce side effects or reduce the therapeutic efficacy, particularly the issues of nonspecific drug release in the absence of ROS and suboptimal release in the presence of ROS. Therefore, the development of novel biomaterials with improved safety profiles and tunable response toward different levels of ROS will undoubtedly help unleash the potential of this field.

The subcutaneously injectable PPS-*bl*-PEG filomicelle hydrogel (FM-depots) represents a promising platform to achieve these goals (*120*). The FM-depots were prepared by physical crosslinking of eight-arm PEG with self-assembled PEG-*bl*-PPS filomicelles. While traditional long-acting hydrogel scaffolds are likely to induce chronic inflammatory responses due to foreign body responses, which lead to patient discomfort and disrupted drug release kinetics (*155*–*157*), subcutaneously injected blank PEG-*bl*-PPS FM-depots induce negligible inflammatory responses within injection sites in both rodents and nonhuman primates (*118*, *120*, *158*–*160*). After single injection of anti-inflammatory 1,25 dihydroxyvitamin D3 (aVD)–loaded PEG-*bl*-PPS FM-depots in atherosclerotic mice, the depots sustainably released aVD-loaded micelles at the injection site in response to homeostatic level of ROS, followed by the accumulation of aVD-loaded micelles in lymphoid organs such as the spleen and lymph nodes. The micelles were then engulfed by APCs and underwent specific ROS-responsive release of aVDs in endosomes of DC, inducing DC tolerance and activating regulatory T cells (T_regs_), which subsequently migrated to atherosclerotic lesions and induced immunosuppression for months (*120*). The good safety profile and controlled release profile of PEG-*bl*-PPS FM-depots make this platform highly attractive for immunomodulatory drug delivery.

Future research is warranted to expand the range of materials available for the ROS-promoted delivery of various therapeutics to target sites at multiple levels (tissue, cellular, and subcellular levels). In-depth mechanistic studies are also needed to guide the use of ROS in conjunction with the administration of therapeutics and the rational choice of therapeutics that are compatible with ROS. For example, while ROS can induce inflammation that can facilitate the accumulation of therapeutics in the target tissue, excessive ROS may substantially damage the blood vessel, potentially compromising therapeutic delivery.

## CONCLUSIONS AND OUTLOOK

Modulating ROS has undoubtedly provided exciting opportunities for treating infectious diseases, cancer, and autoimmune diseases through both ROS-modulating and ROS-based therapeutic delivery strategies. While some ROS-modulating therapies to fight pathogens and cancer have quickly advanced to the clinical stage and acquired regulatory approval (Table 1), applying these therapies across targets, especially in deep tumors such as pancreatic cancer and brain cancer, remains challenging. To address these challenges and enhance the therapeutic efficacy of ROS-based treatments, the field would benefit from novel methods that can efficiently deliver ROS modulators (including inducers and scavengers) to specific targets. Moreover, the development of robust sensitizers that can respond to external stimuli to modulate ROS levels is essential. Furthermore, gaining deeper understanding of the biological effects of various ROS on different cell populations is also essential for guiding the development of innovative ROS-modulating therapies. To bridge the gap between preclinical and clinical studies, it will also be important to establish and validate animal models that can closely mimic key features of human diseases. As modulating ROS has shown great potential for treating infectious diseases, cancer, and autoimmune diseases, we envision that overcoming the above barriers will further unleash the potential of ROS modulation to safely and effectively maximize the therapeutic effect of these promising technologies.
